# The long non-coding RNA GAS5 contributes to the suppression of inflammatory responses by inhibiting NF-κB activity

**DOI:** 10.3389/fphar.2024.1448136

**Published:** 2024-10-09

**Authors:** Debora Curci, Biljana Stankovic, Nikola Kotur, Letizia Pugnetti, Vladimir Gasic, Maurizio Romano, Branka Zukic, Giuliana Decorti, Gabriele Stocco, Marianna Lucafò, Sonja Pavlovic

**Affiliations:** ^1^ Laboratory of Advanced Translational Diagnostics, Institute for Maternal and Child Health IRCCS “Burlo Garofolo”, Trieste, Italy; ^2^ Group for Molecular Biomedicine, Department of Human Molecular Genetics and Genomics, Institute of Molecular Genetics and Genetic Engineering, University of Belgrade, Belgrade, Serbia; ^3^ Department of Life Sciences, University of Trieste, Trieste, Italy; ^4^ Department of Medicine Surgery and Health Sciences, University of Trieste, Trieste, Italy

**Keywords:** lncRNA, Gas5, NF-κB, glucocorticoids, inflammation

## Abstract

**Introduction:**

Nuclear factor kappa B (NF-κB) is a key regulator of immune and inflammatory responses. Glucocorticoid drugs (GC) act through the glucocorticoid receptor (GR) as immunosuppressant also in pediatric patients inhibiting NF-κB activity. The long non-coding RNA GAS5 interacts with the GR, influencing GC activity. No data on the role of GAS5 on GR-dependent inhibition of NF-κB activity have been published.

**Methods:**

This study investigated the impact of GAS5 on NF-κB activity in HeLa cells overexpressing GAS5, both under basal conditions and during GC treatment. The study used EMSA, RNA-immunoprecipitation (RIP), Western blotting, and bioinformatic analyses to assess NF-κB DNA binding, GAS5-p65 interaction, and NF-κB signaling pathway modulation.

**Results:**

GAS5 overexpression increased NF-κB DNA binding activity in untreated cells. RNA-IP confirmed a direct interaction between GAS5 and the NF-κB subunit p65, suggesting a potential regulatory mechanism. GAS5 overexpression led to downregulation of NF-κB target genes, TNF-α, and NR3C1. GC treatment reduced NF-κB DNA binding activity in GAS5-overexpressing cells, indicating a potential synergistic effect. Furthermore, GAS5 overexpression increased IκB levels and reduced p-p65/pan-p65 levels during GC treatment.

**Discussion:**

GAS5 appears to modulate NF-κB activity in a complex manner, influencing both basal and GC-induced signaling. The interaction between GAS5, GCs, and NF-κB is multi-faceted, and further research is needed to fully elucidate the underlying mechanisms. These findings suggest that GAS5 could be a potential target for personalized therapy, particularly in pediatric patients with inflammatory conditions.

## 1 Introduction

Nuclear factor kappa B (NF-κB) plays a central role in regulating immune and inflammatory responses. NF-κB is involved in the development and progression of health conditions associated with chronic inflammation, including type 2 diabetes, obesity, autoimmune disorders, and cancer (Capece et al., 2022). Proinflammatory signals, often conveyed via cytokines, such as tumor necrosis factor (TNF), lead to activation of NF-kB, which is typically a heterodimer of p50 and p65 subunits. In particular, upon receiving proinflammatory signals, IkappaB kinase (IKK) complex phosphorylates IkappaB (IkB), leading to its degradation. NF-κB is released from its inhibitor IkB and translocates to the nucleus, where it binds to DNA, and modulates the expression of target genes (Mulero et al., 2019). In addition, phosphorylation of the NF-κB p-65 subunit by IKK complex and other specific kinases is also essential for activation and fine-tuning of the NF-κB pathway (Christian et al., 2016; Kwon et al., 2016).

Treatment of many inflammatory and malignant diseases relies on glucocorticoid drugs (GC), which act through the glucocorticoid receptor (GR) as anti-inflammatory agents. When activated by GCs, the GR translocates from the cytoplasm into the nucleus and binds, through its DNA binding domain, the GC responsive elements in the regulatory regions of GC responsive genes. The activated GR can also suppress inflammatory signals by directly binding the NF-κB transcription factor, inhibiting its activity (Hudson et al., 2018). In addition to direct binding, the crosstalk between GR and NF-κB can manifest at various levels. This includes competing for binding to specific response element sequences within the promoters of target genes, or competition for a limited pool of coregulators, which may result in the repression of one of these transcription factors (Hudson et al., 2018; Gerber et al., 2021; Giridharan and Srinivasan, 2018).

Long non-coding RNAs (lncRNAs) have emerged as significant regulators of gene expression and hold promise as potential biomarkers for clinical applications. They possess modular domains enabling direct interaction with proteins, including transcription factors like NF-κB, thereby modulating inflammatory responses (Chew et al., 2018), immunity, cell proliferation, and survival (Ruland, 2011). The growth arrest-specific 5 (GAS5) gene encodes a lncRNA that functions as a riborepressor of the GR. Acting as a decoy, GAS5 binds to the GR, inhibiting its ability to recognize binding sites on the DNA of target genes (Kino et al., 2010), thus influencing GC activity and clinical drug response (Ruland, 2011; Gasic et al., 2018; Lucafo et al., 2015; Lucafò et al., 2016). Additionally, GAS5 has been demonstrated to interact with the NF-κB signaling pathway (Ji et al., 2022; Yang et al., 2022; Zhang et al., 2019). However, the precise mechanism of this interaction remains unclear.

Several studies have suggested that GAS5 is implicated in numerous types of cancer, such as leukemia, breast cancer, liver cancer, and lung cancer, as well as autoimmune diseases such as pediatric patients affected by inflammatory bowel disease (IBD) (Ji et al., 2022; Yang et al., 2022; Zhang et al., 2019; Lucafò et al., 2019). Indeed, this lncRNA may play a role in regulating inflammation, but the exact details of this mechanism are not yet fully understood (Lucafò et al., 2019; Xiao and Wang, 2023; Curci et al., 2024). To date, it is unknown if GAS5 has an impact on the GR-dependent repression of NF-κB activity and understanding its role in regulating the GAS5-GR-NF-κB network is of great significance. By understanding the mechanisms involved in this pathway, potential therapeutic targets can be identified and utilized in various diseases associated with inflammation.

The current study explored the influence of lncRNA GAS5 on NF-κB activity in HeLa cell line treated or not with GCs. The aim was to advance current understanding of the complex interplay between these molecular regulators and its role in inflammation.

## 2 Materials and methods

### 2.1 Cell line

The HeLa human cervical carcinoma (ATCC, CCL-2) cell line was grown in DMEM medium (Sigma-Aldrich, Milwaukee, WI, United States) supplemented with 10% FBS, 1% L-glutamine 200 mM, 1% penicillin 10,000 UI/mL and streptomycin 10 mg/mL. Cell culture was maintained according to standard procedures in a humidified incubator at 37 °C and with 5% CO_2_.

### 2.2 Expression studies

Total RNA was extracted from HeLa cells in TRI reagent solution (Ambion Inc., Austin, TX, United States), following the manufacturer’s instructions. The concentration and purity of RNA were measured spectrophotometrically at 260 and 280 nm. cDNA was synthesized from RNA using the RevertAid Reverse Transcriptase (Thermo Fisher Scientific, Waltham, MA, United States), following the manufacturer’s instructions. GAS5 expression levels were quantified by real-time PCR (Applied Biosystems 7,900, Foster city, CA, United States) using the TaqMan gene expression assay kit (Thermo Fisher Scientific, Waltham, MA, United States). To normalize the obtained values, GAPDH gene expression was used as an internal control, using the TaqMan gene expression assay kit (Thermo Fisher Scientific, Waltham, MA, United States). The relative expression levels of GAS5 were calculated using the ΔΔCT method.

### 2.3 Transfection

For GAS5 plasmid transient transfection, Hela cells were seeded in 6-well plates at 3.5 × 10^5^ per well. Twenty-four hours later, cells were transfected with the empty vector or the pcDNA3.1_GAS5 plasmid using Lipofectamine^®^2000 Transfection Reagent (Thermo Fisher Scientific, Waltham, MA, United States) according to the provided protocol. The amount of the plasmid DNA (pcDNA3.1_GAS5 construct and pcDNA3.1 empty) was 4 µg per well. The medium was replaced 5 h after the transfection.

### 2.4 Preparation of total and nuclear extracts

Total and nuclear extracts were prepared using HeLa cells transfected with the empty vector (pcDNA3.1) or the pcDNA3.1_GAS5 plasmid and treated with dexamethasone (DEXA) at the final concentration of 100 nM and 1 µM for 4 and 24 h at 37°C and 5% of CO_2_ on the basis of the data published by Kino T. and colleagues (Kino et al., 2010). To prepare the total lysate 100 μL of a lysis buffer with protease inhibitor cocktail 1× (Thermo Fisher Scientific, Waltham, MA, United States) was used, followed by sonication for 30 s and centrifugation at 10,000 ×g for 10 min. The supernatant contains the whole protein lysate.

To isolate nuclear proteins, cells were collected and resuspended on ice in 400 µL of cold buffer A: 10 mM HEPES [pH 7.9], 10 mM KCl, 0.1 mM EDTA, 0.1 mM EGTA, 1 mM DTT, and 0.5 mM PMSF. Cells were left on ice for 15 min and then 25 µL of 10% NP-40 was added. Samples were vortexed and centrifuged at maximum speed for 30 s. After that, the pellet, which contained cells’ nuclei, was resuspended in 50 µL of cold buffer B (20 mM HEPES [pH 7.9], 0.4 M NaCl, 1 mM EDTA, 1 mM EGTA, 1 mM DTT, and 1 mM PMSF) and left on the shaking platform for 15 min at 4 °C.

The nuclei were collected by centrifugation at 13,000 rpm for 5 min at 4 °C. Concentration of the total and nuclear extract proteins was measured using the Bradford method (Bio-Rad Laboratories, Hercules, CA, United States), following the manufacturer’s recommendations. Nuclear protein extracts were further used for electrophoretic mobility shift assay, while total protein extracts were used for Western blot analysis.

### 2.5 Electrophoretic mobility shift assay (EMSA)

DNA binding activity of NF-κB was analyzed using EMSA. In this method, a radioactively labeled double-stranded probe that contained NF-κB consensus sequence was incubated with nuclear extracts from HeLa cells. After incubation, formed DNA-protein complexes were analyzed using polyacrylamide gel electrophoresis (PAGE) and then their presence was detected by autography.

In EMSA experiments, 21bp length NF-κB probe was used after hybridization of sense and antisense oligonucleotides, from which sense oligonucleotide was previously radioactively labeled. The sequences of the oligonucleotides used for preparing the NF-κB probe were as follows.

• NF-kB_sense 5′- AGT​TGA​GGG​GAC​TTT​CCC​AGG - 3′
• NF-kB_antisense 5’ - CCT​GGG​AAA​GTC​CCC​TCA​ACT - 3′

Ten pmol of NF-κB sense oligonucleotide was labelled at the 5′ end with 1.6 pmol of [γ-32^P^] dATP (32^P^, radioisotope phosphorus-32) using T4 polynucleotide kinase (Thermo Fisher Scientific, Waltham, MA, United States) at 37 °C for 45 min, according to the manufacturer’s instructions. Afterwards, the excess of free radioactive nucleotides was eliminated using Sephadex G-50 columns (Sigma-Aldrich, Milwaukee, WI, United States). Labelled NF-kB_sense oligonucleotide was then completely dried in the vacuum centrifuge and dissolved in 25 µL of the annealing buffer (10 mM Tris-Cl pH 7.5, 50 mM NaCl, 10 mM MgCl_2_, 1 mM EDTA, 1 mM DTT). Fifty pmol of the NF-kB_antisense oligonucleotide was added to the annealing mixture to ensure complete hybridization of the radioactively labelled sense oligonucleotide. Hybridization of the sense and antisense oligonucleotides was acquired after short denaturation at 90 °C for 3 min and renaturation at room temperature.

To enable DNA-protein binding, 3 µg of HeLa nuclear extract were incubated at 37°C for 30 min with 10,000 cpm of NF-κB probe in the presence of 50 ng of double-stranded nonspecific competitor Poly (dI-dC) (Sigma-Aldrich, Milwaukee, WI, United States). The binding reaction was performed in a 25 µL final volume with the 1× binding buffer (5 mM Tris-Cl pH 8.0, 25 mM NaCl, 0.5 mM DTT, 0.5 mM EDTA, 5% glycerol).

The EMSA reaction’s specificity was shown through the addition of a specific competitor (a 10-fold molar excess of cold, unlabeled NF-κB probe) or by using 2 µg of anti-p65 antibody (Santa Cruz Biotechnologies, Dallas, TX, United States).

After incubation, the binding reaction was loaded on the 4% polyacrylamide gel, and electrophoresis was run using 250 V and 65 mA at 16°C for 1 h with 0.5% TBE. After PAGE, gel was dried and DNA-protein complexes were analyzed using Cyclone Phosphor Imager (Perkin Elmer, Waltham, MA, United States) and autoradiography (X-ray-sensitive films were exposed to the dried gels in the cassettes at −80°C for 24 h). Films were scanned in high resolution using transmission scanning and intensities of the formed complexes were quantified densitometrically with ImageJ software (Schneider et al., 2012).

### 2.6 RNA immunoprecipitation (RNA-IP)

HeLa cells lysates were obtained using the RIP buffer, composed of 20 mM HEPES pH 7.7 (Euroclone^®^, Pero (Mi), Italy), 150 mM NaCl, 0.5 mM EDTA, 1 mM DTT, 10% glycerol, 0.1% TritonX (Sigma-Aldrich, Milwaukee, WI, United States) with protease inhibitor cocktail (Thermo Fisher Scientific, Waltham, MA, United States). After an incubation of 30 min in ice, samples were centrifuged at 10,000 ×g for 20 min at 4°C to remove cell debris. The supernatant containing the whole protein lysate was incubated with Sepharose G Beads (1:1) (Sigma-Aldrich, Milwaukee, WI, United States) for 20 min at 4°C, for sample pre-clearing. After, beads were pelleted at 200 ×g for 30 min, and the supernatants were incubated overnight at 4°C in a solution with RIP buffer, 5 μg/mL heparin and the primary antibodies anti-P65 (Santa Cruz Biotechnologies, Dallas, TX, United States), and anti-IgG (Sigma-Aldrich, Milwaukee, WI, United States), as negative control. Sepharose G Beads were added to the samples and incubated for 4 h at 4 °C. At the end, samples were divided in two aliquots and then centrifuged at 1,000 rpm for 30 s. For the first aliquot, used for RNA isolation, the supernatant was removed, and beads were resuspended in 500 μL of RNA wash buffer (HGEN buffer with DOC 0.2% and urea 5 mM), incubated 10 min at 4°C with gentle rotation, then centrifuged for at 5,000 xg for 30 s, and repeated for a total of four washes. Beads were resuspended in TRIzol^®^ reagent (Thermo Fisher Scientific, Waltham, MA, United States). Co-precipitated RNAs were isolated and quantitative reverse transcription PCR (real-time PCR) for GAS5 was performed. The second aliquot was used for Western blot to verify the immunoprecipitation of the protein of interest. Beads were washed three times in the Wash buffer (PBS 0.2% TritonX, all from Sigma-Aldrich, Milwaukee, WI, United States). Beads were then resuspended in Loading Buffer 4X (Thermo Fisher Scientific, Waltham, MA, United States) and protein (P65) was detected by Western blot analysis. The relative enrichment of one RNA-IP experiment was calculated by the ratio between IP mRNA levels and total lysate (INPUT) mRNA and normalized to negative control (anti-IgG).

### 2.7 Western blotting

Total cell extracts were prepared in RIPA buffer without SDS (150 mM NaCl, 50 mM Tris-HCl pH 8.0, 1 mM EDTA, 1% NP-40, 0.5% Na-deoxycholate) supplemented with protease inhibitor cocktail 1X (Thermo Fisher, Waltham, MA, United States). After sonication, protein concentrations were determined by BCA assay using bovine serum albumin as standard. Twenty μg of lysates were loaded on Sodium Dodecyl Sulphate–Polyacrylamide Gel Electrophoresis (SDS-PAGE) 4–12% gradient gels (NUPAGE 4%–12%, Bis-Tris Plus Gels, Thermo Fisher, Waltham, MA, United States) and transferred, in wet condition, onto a nitrocellulose membrane.

Afterwards, each sample was incubated with 5% milk solution in T-TBS (10 mM Tris-HCl pH 8, 150 mM NaCl; 0.1% Tween 20) for 1 h at 4°C on a rocking platform. After blocking, incubation with the following primary antibodies was performed overnight, at 4 C: anti p65 mouse 1:500 (Santa Cruz Biotechnologies, Dallas, TX, United States), anti-phospho p65 rabbit 1:1,000 (Ser536) (Cell Signaling Technologies, Danvers, MA, United States), anti-IΚB rabbit 1:3,000 (Abcam, Cambridge, United Kingdom) anti-β-actin mouse 1:3,000 (Abcam). Anti-mouse (Cell Signaling Technologies, Danvers, MA, United States) and anti-rabbit HRP conjugated (OriGene Technologies, Rockville, MD, United States and Sigma-Aldrich, Milwaukee, WI, United States) secondary antibodies were incubated for 1 h at 4 °C. Proteins were detected by chemiluminescence using a ChemiDoc system (Bio-Rad Laboratories, Hercules, CA, United States) and the band’s density was analyzed using the ImageJ program. All data were normalized on β-actin.

### 2.8 Bioinformatics analysis

To investigate the potential interactions between GAS5 and RELA (p65), the NPInter database (http://bigdata.ibp.ac.cn/npinter5/) (Zheng et al., 2023), which integrates RNA-protein interactions derived from various high-throughput data sources, was used. This analysis allowed us to explore the functional interaction between GAS5 and p65, providing evidence for a direct binding interaction between these molecules.

Additionally, to explore the role of RNA-binding proteins (RBPs) in facilitating the interaction between GAS5 and RELA, the ENCORI database (https://rnasysu.com/encori/) (Li et al., 2014), which offers comprehensive data on RNA-binding protein interactions based on CLIP-seq data was used. We retrieved the RBPs interacting with GAS5 from this database. To complement this analysis, the IntAct database (https://www.ebi.ac.uk/intact/) (Del Toro et al., 2022), a repository and analysis platform for molecular interaction data was used. We specifically interrogated this database to identify proteins interacting with p65. By overlapping the sets of RBPs interacting with GAS5 and p65, we identified shared RBPs that may mediate or stabilize the GAS5-p65 interaction.

### 2.9 Statistical analysis

Statistical analyses were performed using Graph-Pad Prism version 4.00 (GraphPad, La Jolla, CA, United States). Wilcoxon sign rank test was used for the EMSA analysis. T-test was used to analyze Western blot assay results and gene expression. *p*-values <0.05 were considered statistically significant.

## 3 Results

### 3.1 Effect of GAS5 on NF-κB DNA binding activity

We examined the effect of overexpressed GAS5 on NF-κB DNA binding activity in transfected HeLa cells using EMSA. The efficiency of the overexpression of the lncRNA in HeLa cells was assessed by real-time PCR after 4 and 24 h (Supplementary Figure S1). Additionally, we stimulated non-transfected HeLa cells with the proinflammatory cytokine TNF-α as a positive control for NF-κB signaling activation. EMSA showed nuclear protein:DNA complexes whose specificity was confirmed with competitive and supershift (anti-p65) assays (Supplementary Figure S2).

**FIGURE 1:**
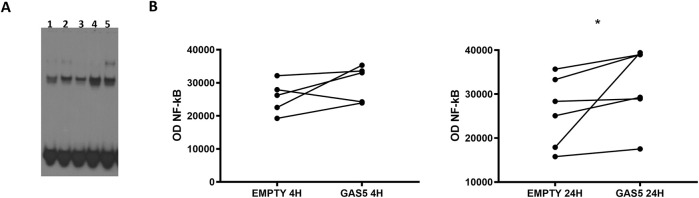
**(A)** Representative NF-κB EMSA analyses in HeLa cells transfected with empty pcDNA3.1 (lanes 1, 3) and pcDNA3.1_GAS5 (lanes 2, 4), after 4 (lanes 1, 2) and 24 h (lanes 3, 4) from transfection or treated with TNF-α (lane 5). EMSA experiments were performed in five to six replicates. **(B)** The optical density (OD) of the shifted bands was measured in all samples. Wilcoxon signed rank test, GAS5 vs. EMPTY 4H *p*-value = 0.187; GAS5 vs. EMPTY 24H *p*-value = 0.031.

In HeLa cells transfected with pcDNA3.1_GAS5, we observed increased binding of NF-κB to DNA consensus probe compared to HeLa cells transfected with empty pcDNA3.1 (Figures 1A, B). A similar effect was observed in TNF-α-induced NF-κB activation in non-transfected HeLa cells. Our findings suggested GAS5 involvement in regulating the NF-κB pathway, enhancing the binding of this transcriptional factor to DNA.

### 3.2 Analysis of GAS5 and NF-κB interaction

**FIGURE 2:**
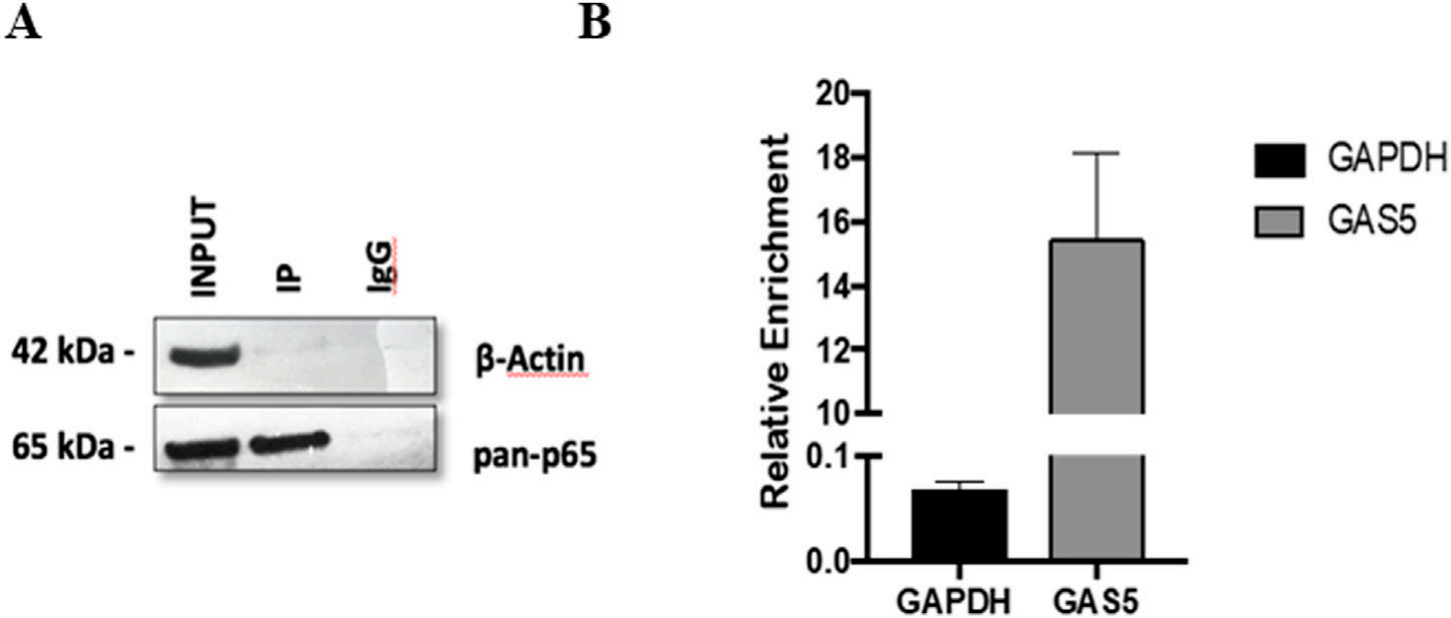
The protein-mRNA immune-complex with either anti-p65 or the control anti-IgG complexes were processed for immunoblotting **(A)** and for Real Time-PCR **(B)**. Total lysate (INPUT) was not subjected to immunoprecipitation. GAPDH mRNA was used as a control for RNA-IP specificity. The relative enrichment for GAS5 or GAPDH mRNA, reported in the graph, was calculated by the ratio between IP anti-p65 mRNA and INPUT mRNA (ΔCt = Ct_IP - Ct_Input), and normalized to negative control (IP anti-IgG) mRNA (ΔΔCt = ΔCT_IP - ΔCt_IgG. Relative enrichment, RE = 2^−ΔΔCT^. Three experiments were conducted in total. WB image was from one representative experiment.

The potential interaction between the lncRNA GAS5 and the NF-κB subunit p65 was further investigated using an RNA immunoprecipitation (RNA-IP) experiment in HeLa cells. The specificity of the immunoprecipitation assay was confirmed by the observation that the p65 protein was successfully isolated using a specific antibody (anti-p65) (Figure 2A, lane IP) but not using an IgG control antibody (anti-IgG) (Figure 2A, lane IgG). Subsequently, the presence of GAS5/p65 interaction was tested by RNA-IP followed by Real-Time PCR targeting GAS5 or the housekeeping gene GAPDH, to confirm the specific enrichment of the assay. The result clearly showed that GAS5 was present in the protein complex with p65, indicating a potential direct physical bond (Figure 2B).

### 3.3 In silico analysis of GAS5-p65 interaction

To explore the interaction between GAS5 and the NF-κB subunit p65/RELA, we carried out a series of bioinformatic analyses. Utilizing the NPInter database, which compiles RNA-protein interaction data from high-throughput sequencing techniques such as CLIP-seq, we identified a significant RNA-protein interaction between GAS5 and p65. This interaction, classified as a binding event (ID: ncRI-50112523), was detected in a CLIP-seq dataset (GSE197707), suggesting that GAS5 directly binds to p65. The functional annotation of this interaction indicates that GAS5 may play a role in the positive regulation of I-kappaB kinase/NF-kappaB signaling, implying a potential regulatory influence of GAS5 on NF-κB pathways through its interaction with p65.

**FIGURE 3:**
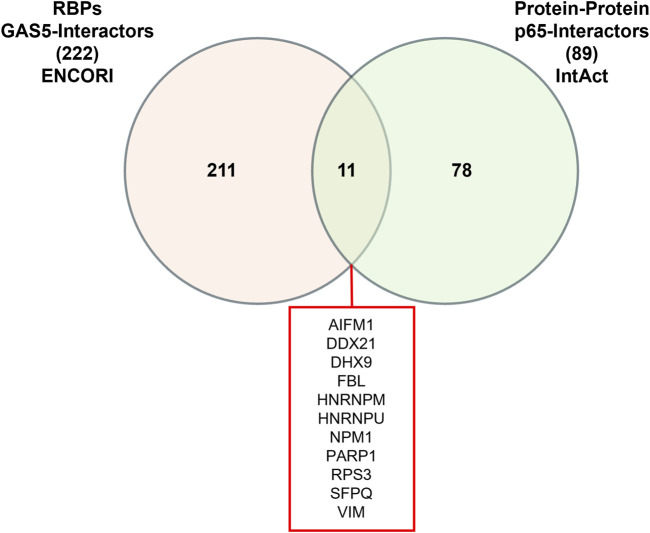
Venn Diagram of RNA-binding proteins (RBPs) interacting with GAS5 and p65.

Given that RNA-protein interactions can be mediated or stabilized by RNA-binding proteins (RBPs), we further investigated the potential involvement of RBPs in the GAS5-p65 interaction. From the ENCORI database, we retrieved 222 RBPs that interact with GAS5. Similarly, the IntAct database provided a list of 89 RBPs that interact with RELA. Venn analysis identified 11 RBPs that are common interactors of both GAS5 and RELA, including AIFM1, DDX21, DHX9, FBL, HNRNPM, HNRNPU, NPM1, PARP1, RPS3, SFPQ, and VIM (Figure 3).

### 3.4 Effects of GAS5 on NF-κB target genes’ expression

**FIGURE 4:**
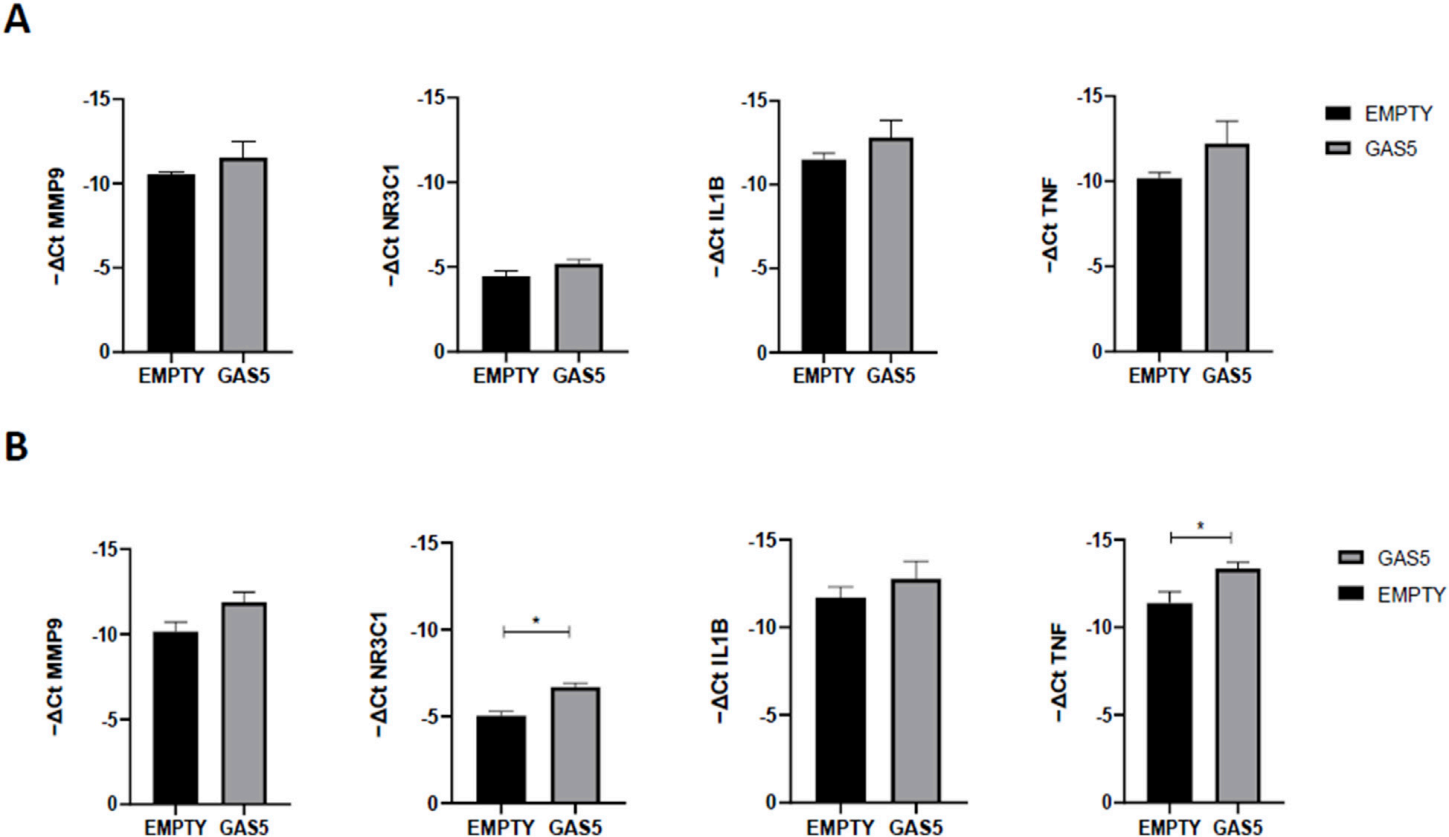
MMP9, NR3C1, IL1B, and TNF gene expression (-ΔCt) in HeLa cells transfected with pcDNA3.1_GAS5 (GAS5) or the empty pcDNA3.1 vector (EMPTY), after 4 **(A)** and 24 **(B)** hours. Gene expression was normalized using the expression of GAPDH gene. Unpaired t-test GAS5 vs. EMPTY at 4h and 24 h *p < 0.05, **p < 0.01. The data are reported as means ± SD of three independent experiments performed in triplicate.

To further test the ability of GAS5 to modulate NF-κB activity, we compared the gene expression levels of several NF-κB target genes in GAS5-overexpressed cells (Figure 4). As shown in Figure 4A, no mRNA level changes were observed after 4 h in GAS5-overexpressing cells. After 24 h of transfection, NR3C1 and TNF were significantly downregulated when GAS5 was overexpressed in HeLa cells (Figure 4B).

### 3.5 Effects of GAS5 on GC-induced modulation of NF-κB DNA binding activity

**FIGURE 5:**
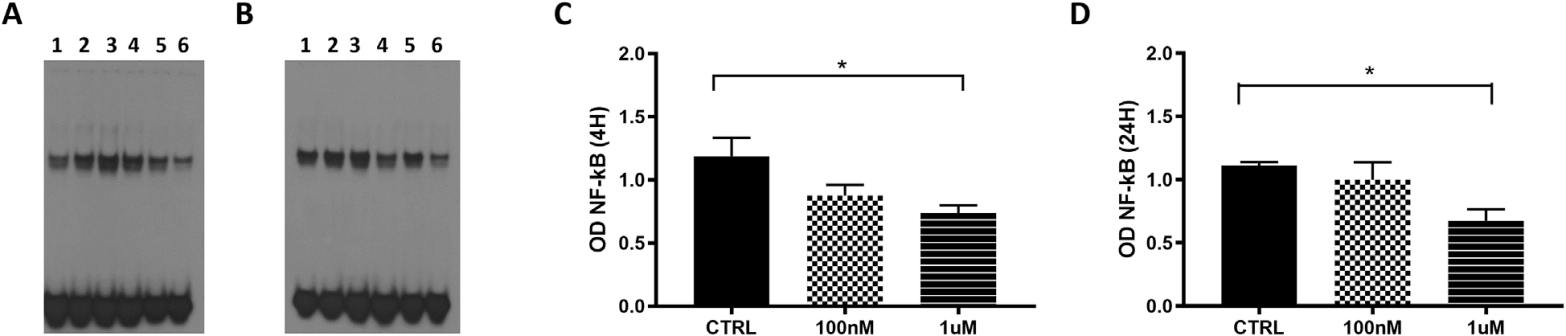
NF-κB EMSA analyses in HeLa cells transfected with empty pcDNA3.1 (lanes 1, 3 and 5) and pcDNA3.1_GAS5 (lanes 2, 4 and 6), treated with DEXA 100 nM (lanes 3 and 4), DEXA 1 µM (lanes 5 and 6) and untreated (CTRL, lines 1 and 2) after 4 h **(A)** and 24 h **(B)**. Nuclear extracts were prepared in triplicate. The EMSA produced optical densities (OD) of the shifted NF-κB bands were measured in all samples after 4 **(C)** and 24 h **(D)**. OD values of pcDNA3.1_GAS5 CTRL, DEXA 100 nM and DEXA 1 µM have been normalized by the OD value of corresponding empty pcDNA3.1. Kruskal–Wallis test, 4 h *p*-value = 0.036; 24 h *p*-value = 0.041; Dunn’s Multiple Comparison Test *p < 0.05.

Using EMSA, we evaluated the effect of overexpressed GAS5 on NF-κB DNA binding activity in transfected HeLa cells during the treatment with the GC dexamethasone (DEXA) at 100 nM and 1 µM for 4 and 24 h. We observed differences in the level of NF-κB DNA binding activity within samples measured at 4 h under control, 100 nM and 1 µM conditions in HeLa cells transfected both with empty and pcDNA3.1_GAS5 vector (*p* = 0.049 and *p* = 0.038, respectively), however, after *post hoc* pairwise comparisons, we found no differences between these conditions (Supplementary Figure S3). However, when observed relative to the cells transfected with the empty plasmid, in the cells transfected with pcDNA3.1_GAS5 construct, NF-κB DNA binding activity was decreased in response to DEXA treatment: statistically significant for 1 μM, but not for 100 nM DEXA treatment. The same trend was observed after both 4 and 24 h of DEXA treatment (Figure 5).

### 3.6 Effect of GAS5 on the p65 activation during GC treatment

**FIGURE 6:**
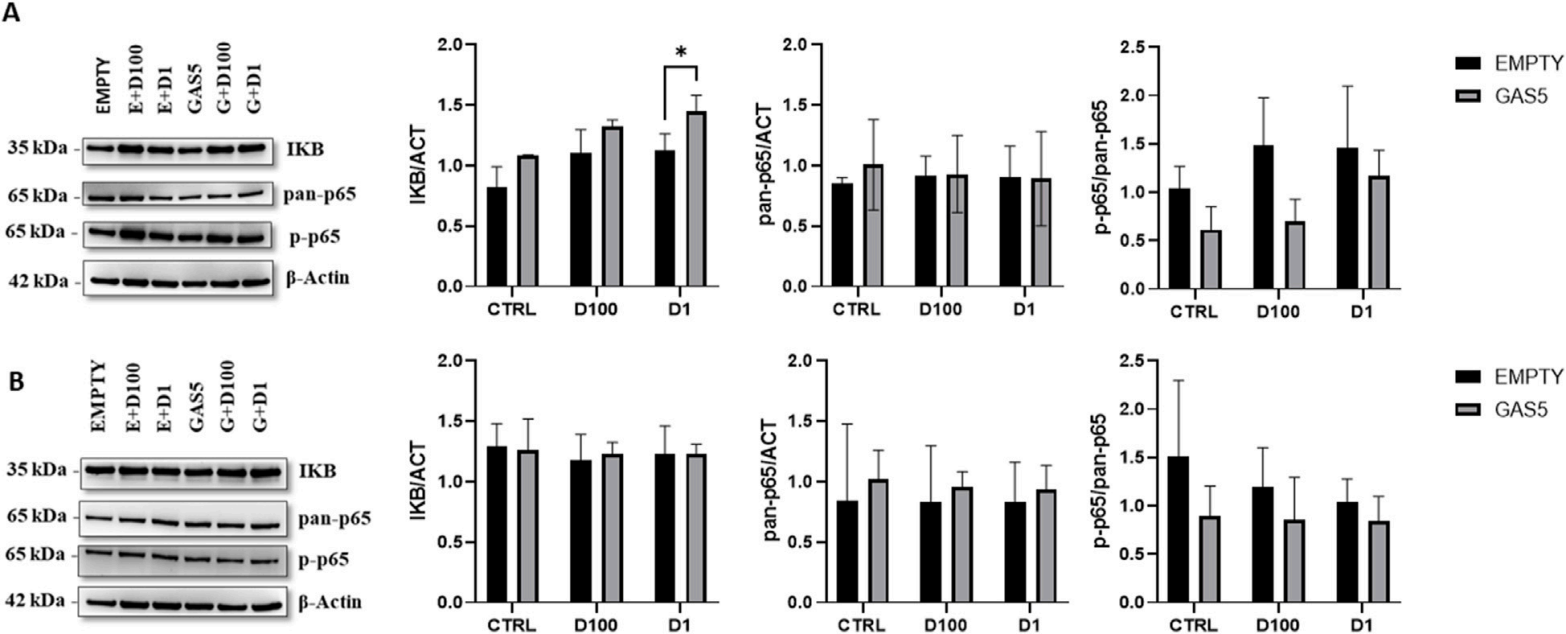
Representative Western blot and quantification histogram for protein levels of IKB, pan-p65, and p-p65 in untreated cells (CTRL) after 4 panel **(A)** and 24 h Panel **(B)** of treatment with 100nM and 1 μM of dexamethasone (D100 and D1). β-actin was used as internal control. The levels of p-p65 expression were expressed as p-p65/pan-p65 ratio. Panel A, 2-way ANOVA IKB/ACT: Row Factor (Treatment condition): *p* = 0.002, Column Factor (Empty and GAS): *p* = 0.001. Sidak’s multiple comparisons test: *p < 0.05; pan-p65/ACT: Row Factor (Treatment condition): p = ns, Column Factor (Empty and GAS): p = ns, p-p65/pan-p65: Row Factor (Treatment condition): p = ns, Column Factor (Empty and GAS): *p* = 0.017. Panel B, 2-way ANOVA IKB/ACT: Row Factor (Treatment condition): p = ns, Column Factor (Empty and GAS): p = ns; pan-p65/ACT: Row Factor (Treatment condition): p = ns, Column Factor (Empty and GAS): p = ns, p-p65/pan-p65: Row Factor (Treatment condition): p = ns, Column Factor (Empty and GAS): p = ns. The data are reported as means ± SD of three independent experiments performed in triplicate.

To evaluate whether the effect of overexpressed GAS5 during GC treatment could alter p65 activation, we measured IKB levels, total p65 (pan-p65), and phospho-p65 (p-p65) levels in HeLa cells by Western blot. Levels of IKB protein increased significantly after 4 h of DEXA treatment in HeLa cells, particularly in GAS5-overexpressing cells (Figure 6A). Total p65 (pan-p65) did not change in cells after 4 h of GC incubation (Figure 6A). Furthermore, overexpression of GAS5 seems to reduce p-p65/pan-p65 ratio when compared to cells transfected with empty plasmid (Figure 6A). No significant change in protein levels after 24 h of DEXA treatment was observed for all proteins analyzed (Figure 6B).

## 4 Discussion

Dysregulation of NF-κB has been implicated in various inflammatory and autoimmune diseases, as well as cancer (Mao et al., 2017). Activity of NF-κB signaling pathway is regulated by several factors including lncRNAs. For instance, NKILA and Lethe are lncRNAs known to impact NF-κB activity by inhibiting its DNA binding ability (Ghafouri-Fard et al., 2021; Liu et al., 2015). While several studies have investigated the interplay between the lncRNA GAS5 and NF-κB-related pathways, the precise regulatory mechanisms by which GAS5 affects NF-κB remain unclear. Our study, for the first time, showed increased DNA binding activity of NF-κB in HeLa cells overexpressing GAS5, confirming its involvement in regulating the NF-κB pathway. Moreover, the RNA-IP result highlighted the ability of GAS5 to bind p65 subunit and the identification of this interaction through the NPInter database, along with its classification as a binding interaction in CLIP-seq datasets, reinforces the hypothesis that GAS5 plays a regulatory role in the NF-κB signaling cascade. To gain deeper insights into the mechanisms underlying this interaction, our study also investigated the structural aspects of both GAS5 and p65. The Rel Homology Domain (RHD) of p65, known for its DNA-binding capability, may also have RNA-binding potential. The structural features of the RHD, particularly the immunoglobulin-like beta barrel subdomains, suggest a possible role in RNA recognition (Kaur et al., 2022; Lecoq et al., 2017). Although direct experimental evidence for p65’s RNA-binding domain specifically recognizing GAS5 remains to be confirmed, there is substantial evidence supporting the interaction of NF-κB and its subunits with other lncRNAs (Shin et al., 2024). Future studies focusing on mutagenesis of the RHD will be essential to map the specific regions involved in RNA binding.

Moreover, our *in silico* identification of 11 RBPs that interact with both GAS5 and p65 opens new avenues for understanding the potential indirect interaction mechanisms. These RBPs could act as molecular bridges, facilitating or stabilizing the GAS5-p65 complex, thereby enhancing the regulatory effects of GAS5 on NF-κB signaling. Experimental validation through RBP knockdown or pull-down assays will be critical in determining the roles these proteins play in the GAS5-p65 interaction.

GAS5 is known to have a modular structure, with distinct regions that form specific secondary structures, which may be crucial for its interaction with proteins (Frank et al., 2020). Based on the available secondary structure of GAS5, considering that the 5′ end has low secondary structure content, both the core module (which is highly structured and mediates the effects of mammalian target of rapamycin inhibition on cell growth) and the 3′ end (which contains the steroid receptor binding module) are more likely to interact with proteins other than those already described (Frank et al., 2020).To elucidate the consequence of GAS5-p65 interaction, we tested gene expression of NF-κB target genes in overexpressing GAS5 cells and found downregulation of TNF-α, and NR3C1 after 24 h. These results could be in line with previous data published by Yang and colleagues, in which overexpression of GAS5 led to a reduction in the expression level of proinflammatory genes IL1β, IL6, and IL8 (Yang et al., 2022). Moreover, previous data have also shown that GAS5 overexpression is associated with a lower expression of TNFα, IL-1, IL-6, and IL-8 (Li et al., 2018). Interestingly, in our study NR3C1 is downregulated in overexpressing GAS5 cells. Even in the absence of GC, cytosolic GR has been shown to interact with p65, p50, and IκB, and inhibit nuclear translocation of NF-κB (Widén et al., 2003). Therefore, the observed decrease in GR mRNA upon GAS5 overexpression suggests that GAS5 may influence the mutual antagonism between GR and NF-κB and may be an important basis for GC resistance. These findings highlight the need for further investigation into the mechanisms by which GAS5 affects GR and NF-kB activity and its broader implications in therapeutics (Schaaf and Cidlowski, 2002).

Although the increased DNA binding of NF-kB should theoretically promote its transcriptional activity, the complexity and lack of knowledge about the mechanism of action of GAS5 and its interaction with NF-kB, cannot exclude the possibility that several co-regulators may influence the effects observed. The NF-κB family comprises five activating and repressive subunits that form functional homo- and heterodimers. In particular, the dimers containing the C-terminal transactivation domain (TAD) are capable of recruiting transcriptional co-activators, which promote gene expression; dimers without TADs act as transcriptional repressors (de Jesús and Ramakrishnan, 2020). The interaction between GAS5 and p65 could influence the dimer composition inhibiting the NF-kB transcriptional activity and further investigation should evaluate this possibility. Analyzing total protein extracts in GAS5 overexpressed compared to control HeLa cells, we showed diminished p-p65/pan-p65 levels and increased IκB expression. It has been reported that high levels of GAS5 reduced p-p65 expression, enhanced the level of IκB, and inhibited the entry of p65 into the nucleus within the TNF-α activated NF-κB pathway in periodontal ligament stem cells (Yang et al., 2022). Specific phosphatases could reduce p-p65 levels in GAS5 overexpressed cells (Cai et al., 2002; Tsuchiya et al., 2017), although the role of GAS5 in the regulation of phosphatases such as serine/threonine phosphatase (Protein phosphatase 2A) or Protein phosphatase one is currently unknown.

Interestingly, GAS5 was found bound to IKK and was positively related to IKK and negatively related to NF-κB activity in mouse mesangial cells (Zhang et al., 2019). These results could partly explain the mechanisms underlying the increase in IκB in GAS5-overexpressing cells: the inhibition of IKK activation by the lncRNA could favour the IκB accumulation by limiting its ubiquitin-mediated proteasomal degradation. It will therefore be important to assess the IKK levels and the ability of p65 to enter into the nucleus in our experimental model. We can hypothesise that the decrease in phosphorylation of p65 and its increase in DNA binding are independent and sequential events: GAS5 may rapidly inhibit the phosphorylation of p65, thereby affecting its activation and nuclear translocation, and subsequently alter its DNA-binding capacity. Future studies should focus on determining their temporal dynamics to fully understand how GAS5 modulates NF-κB signaling.

We further analyzed the effects of GAS5 on the NF-κB activity under GC treatment. In our EMSA, DEXA treatment reduced the ability of the transcription factor NF-κB to bind DNA in HeLa cells overexpressing GAS5. Downregulation of NF-κB DNA binding activity by GCs has been described in various studies (Auphan et al., 1995; He et al., 2017; Mukaida et al., 1994; Scheinman et al., 1995). To date, it is unknown if GAS5 could impact the GR-dependent repression of NF-κB activity, but it is already known that GAS5 binds to GR at the same domain, of which GR could interact with NF-κB (De Bosscher et al., 2003). Since GAS5 acts as a regulatory repressor of the GR, one would expect diminished negative regulation of NF-κB-mediated transcription by DEXA in our model system. However, we observed a decrease in NF-κB DNA binding activity, suggesting the existence of possible alternative regulatory mechanisms. Decrease of NF-κB DNA binding activity in GAS5 overexpressed conditions during DEXA treatment could reflect displacement of NF-κB from its response elements or decreased activation of NF-κB in cytoplasm and its translocation to the nucleus. However, additional experiments should be performed to explain and confirm this mechanism.

Next, we explored whether GAS5 could modulate the protein expression of IκB and p65 during treatment with GCs. At 4 h of DEXA treatment, a significant increment of IκB protein after GC treatment in HeLa cells was found, as already reported in other studies (Auphan et al., 1995; Scheinman et al., 1995; He et al., 2020) where the increased expression of IκB has been proposed as the principal mechanism for the immunosuppressive GC effect (Newton et al., 1998). In our study, GC-related increase of IκB levels was higher in HeLa cells overexpressing GAS5 following treatment with DEXA, which implies that GAS5 may play a regulatory role in modulating the interaction between GCs and NF-κB. It is possible that GAS5 promotes the effects of GCs on IκB by reducing NF-κB activity. Additionally, the active form of p65 was lower in GAS5-overexpressing cells respect to the control. The phosphorylation of p65 subunit is necessary for cytoplasmic to nuclear localization of NF-κB/p65 and downstream target genes transcription (Christian et al., 2016). When inactive, NF-κB is bound to IκBα, which masks the nuclear localization signal of p65 reducing the rate of shuttling of NF-κB between the nucleus and cytoplasm. Interestingly, it has been shown that the exposure to GCs can increase NF-κB translocation to the nucleus, indicating that the effects of GCs on NF-κB pathway are environment-dependent and not related strictly to inhibition of NF-κB activation (Feng et al., 2019; Jong-Hun et al., 2019). In this context, the IκB and p-p65 levels during GAS5 overexpression in HeLa cells could not explain the decrease in DNA binding activity of NF-κB under GC treatment. Therefore, we hypothesize that mechanisms of GAS5 interference with the NF-κB pathway could be orchestrated independently of the GC pathway through potential direct interaction with p65, as demonstrated from the RIP experiments. However, further analysis of cells transfected with GAS5 in presence of a steroid treatment and after an inflammatory stimulation, such as TNF-α, should be performed to better understand the implication of NF-κB interaction.

In conclusion, our study showed that GAS5 presents distinct modulatory roles on the NF-κB pathway under basal and GC treatment conditions. GAS5 increases NF-κB DNA binding activity in non-treated cells, while decreasing it in GC-treated cells. The lncRNA GAS5 seems to physically interact with a member of the NF-κB family, p65, and influences the expression pattern of NF-κB-target genes. These results indicate that GAS5 could be considered a regulator of the NF-κB signaling pathway. Interaction between GAS5, GCs, and NF-κB is complex and probably involves more than one regulatory mechanism and further studies are therefore necessary. These regulatory mechanisms are most probably cell specific and should be examined in different conditions, such as models of diseases associated with dysregulated inflammatory responses, cell survival and apoptosis or poor GC therapeutic effects. These results could be useful for developing strategies to personalise therapy, particularly for pediatric patients.

## Data Availability

The original contributions presented in the study are included in the article/Supplementary Material, further inquiries can be directed to the corresponding author.
