# Supplementary material for: The long non-coding RNA GAS5 contributes to the suppression of inflammatory responses by inhibiting NF-κB activity
Source: Front Pharmacol. 2024 Oct 9;15:1448136. doi: 10.3389/fphar.2024.1448136 (PMC11496153; doi:10.3389/fphar.2024.1448136)
Supplement: Supplementary file 1 [file DataSheet1.docx]

Supplementary Material

## Supplementary Figures


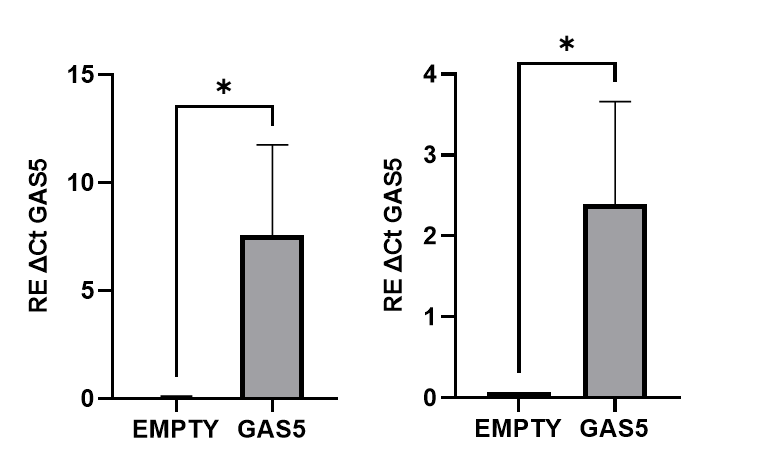


A

B

**Supplementary Figure 1.** Evaluation of GAS5 levels after transfection with pcDNA-GAS5 plasmid in Hela cells after 4 (Panel A) and 24 hours (Panel B). GAS5 expression was normalized using GAPDH gene and relative expression (RE) values are expressed as 2^−ΔCt^. Unpaired t test * p < 0.05. The data are reported as means ± SD of three independent experiments performed in triplicate.


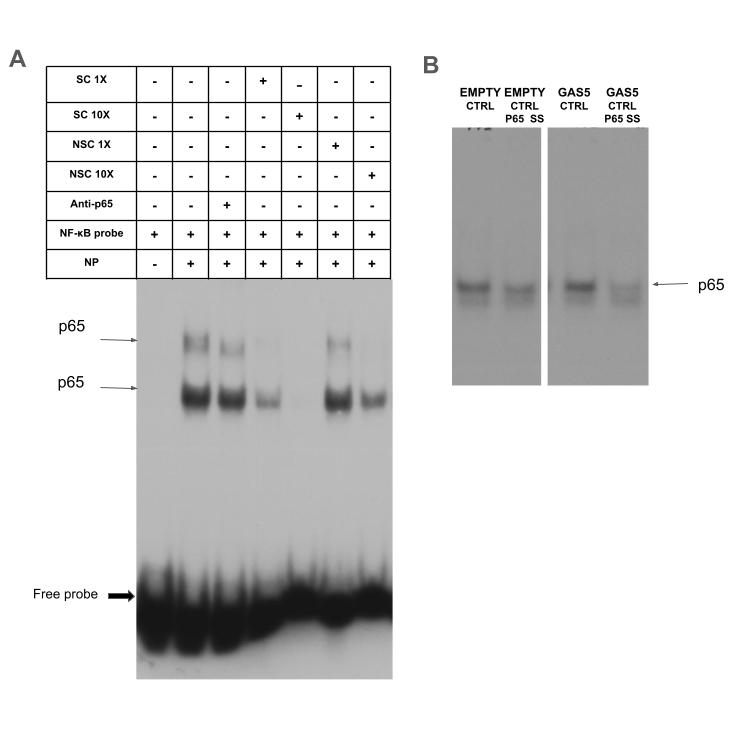
**Supplementary Figure 2.** Competitive and supershift EMSA. (A) EMSA shows binding of 3 µg of untransfected HeLa cells nuclear proteins to 32P labeled DNA probes containing NF-κB consensus sequence. For competitive assays, an unlabeled NF-κB DNA probe (specific competitor) was used in 1X and 10X molar excess compared to labeled NF-κB probe. An unlabeled probe containing NF-κB binding unrelated sequence was used as a non-specific competitor in 1X and 10X molar excess compared to labeled NF-κB probe. For supershift assay, 2 µg of anti-p65 antibody was used to demonstrate presence of p65 in protein-DNA complexes. (B) Anti-p65 supershift assay was assessed in EMPTY and GAS5 CTRL transfected cells (3 µg of NP, 2 µg anti-p65 antibody) (B). SC - specific competitor, NSC - non-specific competitor, NP - nuclear proteins, SS – supershift.


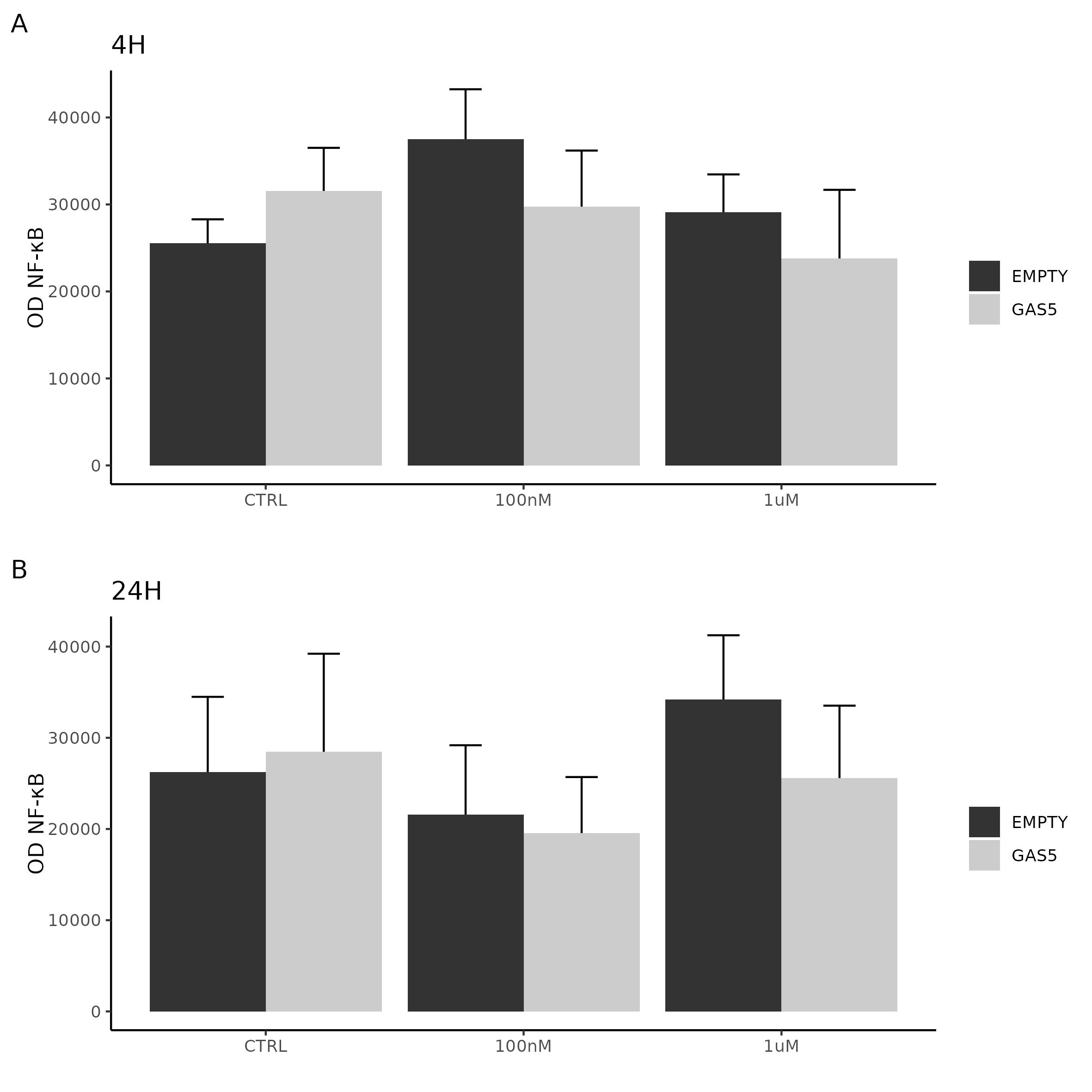


**Supplementary Figure 3.** Level of NF-kB DNA binding activity in HeLa cells transfected with EMPTY or GAS5-constructed pcDNA3.1 vector and treated with dexamethasone (100 nM and 1 µM) for 4H (A) and 24H (B). Level of NF-kB DNA binding activity was determined using EMSA and presented in optical density units (OD) using ImageJ. Differences within samples in different treatment groups (control, 100 nM, 1 µM) were assessed using a non-parametric Friedman test for repeated measurements, followed by post-hoc pairwise comparisons by Wilcoxon sign-rank test and Benjamini–Hochberg multiple comparison correction: (A) Friedman test EMPTY 4H, CTRL vs 100 nM vs 1 µM, p=0.049, no significant difference between treatments and control after pairwise comparisons; Friedman test GAS5 4H, CTRL vs 100 nM vs 1 µM, p=0.038, no significant difference between treatments and control after pairwise comparisons. (B) Friedman test EMPTY 24H, CTRL vs 100 nM vs 1 µM, p=1; Friedman test GAS5 24H, CTRL vs 100 nM vs 1 µM, p=0.37.
